# Frailty and liver transplant eligibility beyond MELD scores: integrating functional status into candidate selection

**DOI:** 10.1186/s12876-025-04333-9

**Published:** 2025-10-21

**Authors:** Maha Elsabaawy, Sameh Afify, Warda Othman, Amr Ragab

**Affiliations:** Department of Hepatology and Gastroenterology, National Liver Institute, Menoufia University, Shebeen El-Kom, Menoufia Egypt

**Keywords:** Liver transplantation, Frailty, Clinical frailty scale, MELD, Transplant eligibility

## Abstract

**Background:**

The MELD score, while central to liver transplant prioritization, fails to capture frailty, an important predictor of transplant eligibility and outcomes.

**Aim:**

To assess the impact of frailty on transplant eligibility and compare its predictive accuracy to MELD-Na.

**Methods:**

We prospectively studied adults with cirrhosis referred to for transplant evaluation. Frailty was assessed using the Clinical Frailty Scale (CFS) by a multidisciplinary team, with adjudication for disagreements. Laboratory data were collected, MELD scores calculated, and transplant eligibility determined using our institutional framework. Correlation, logistic regression, and ROC analyses compared MELD and CFS in predicting eligibility.

**Results:**

Among 672 patients evaluated, 76 (11.3%) met the primary endpoint of transplant eligibility. Eligible patients had significantly lower MELD scores, but better frailty status compared with ineligible patients (mean CFS 3.2 vs. 5.8, *p* < 0.001). CFS correlated moderately with MELD (*r* = 0.62) and showed superior predictive accuracy for eligibility (AUC 0.84 vs. 0.72).

**Conclusion:**

Frailty, assessed by the Clinical Frailty Scale, outperformed MELD-Na in predicting transplant eligibility and proved relevant even in low-MELD populations. These findings highlight frailty as a practical determinant of eligibility and a step toward standardized, evidence-based equitable selection frameworks.

## Introduction

Orthotopic liver transplantation (OLT) remains the definitive treatment for end-stage liver disease, yet the persistent gap between organ availability and demand necessitates precise and equitable candidate selection [1]. The Model for End-Stage Liver Disease (MELD) score, while widely adopted as a tool to prioritize transplant allocation, is fundamentally a reflection of hepatic biochemical dysfunction [2]. It does not account for key extrahepatic factors, most notably, functional status and physiological reserve that critically influence both pre- and post-transplant outcomes [3].

Frailty, characterized by diminished physiological resilience and increased vulnerability to stressors, has emerged as a robust predictor of adverse outcomes across medical disciplines, including surgery and critical care [4]. In the context of liver transplantation, frailty has been independently associated with higher waitlist mortality, postoperative complications, prolonged hospitalization, and reduced graft survival [5]. Despite growing recognition of its prognostic relevance, frailty is not yet systematically integrated into transplant eligibility criteria, and its interaction with MELD-based allocation systems remains poorly defined.

Previous studies have highlighted the prognostic value of various frailty indices, but many were retrospective, lacked standardized assessments, or did not directly evaluate frailty’s role in eligibility decision-making [6]. Moreover, few investigations have specifically addressed the predictive power of frailty relative to MELD in determining the eligibility for transplantation, particularly within resource-constrained or high-burden transplant systems [7].

Therefore, this prospective study aims to evaluate whether frailty, assessed using the Clinical Frailty Scale (CFS), independently predicts liver transplant eligibility among patients with cirrhosis, and to compare its predictive performance to that of the MELD score. By doing so, we seek to establish the clinical value of integrating frailty assessment into the transplant evaluation process.

## Methods

### Study design and setting

We conducted a prospective observational cohort study at the National Liver Institute, Menoufia University, a tertiary referral center for liver transplantation. The study period extended from September 15, 2024, to May 15, 2025. Ethical approval was obtained from the Institutional Review Board (IRB No. 0014014FWA00034015), and all participants or their legal representatives provided written informed consent.

### Participants

All consecutive adult patients (≥ 18 years) with cirrhosis referred to as liver transplant evaluation during the study period were screened. Cirrhosis was diagnosed using histology or a combination of clinical, biochemical, and radiological features in accordance with AASLD guidelines [8].

### Inclusion criteria

- Adults with cirrhosis undergoing liver transplant evaluation.
- Complete clinical and frailty assessment data available.

### Exclusion criteria

- Acute liver failure without underlying cirrhosis.
- Candidates for multi-organ transplantation.
- Incomplete or missing frailty assessment.
- Absolute contraindications (e.g., uncontrolled sepsis, severe cardiopulmonary dysfunction, active malignancy).
- Extra-MELD indications (e.g., hepatopulmonary syndrome, refractory ascites, cholestatic pruritus, hepatoblastoma). We restricted our cohort to patients listed for liver transplantation based on MELD-driven indications. This decision was made to reduce heterogeneity and ensure that eligibility determinations were primarily driven by MELD scores, thereby allowing us to isolate the relative contribution of frailty in the context of MELD-based allocation.

After exclusions, 672 patients were enrolled.

This decision was made to reduce heterogeneity and ensure that eligibility determinations were primarily driven by MELD scores, thereby allowing us to isolate the relative contribution of frailty in the context of MELD-based allocation.

While our institutional framework generally considers a MELD score ≥ 15 as a benchmark for transplant eligibility, final eligibility is determined through multidisciplinary committee consensus. This process accounts for both hepatic severity and extrahepatic factors such as frailty, recurrent decompensation, refractory complications, and quality of life impairments. As such, select patients with MELD scores < 15 was deemed eligible when other clinical indicators warranted transplantation despite lower biochemical severity.

### Eligibility criteria and weighting system

Eligibility was determined by a multidisciplinary transplant selection committee using a standardized, weighted scoring framework designed to incorporate both hepatic severity and extrahepatic factors. Each domain contributed a specific proportion to the overall eligibility score:

- MELD score ≥ 15 (30%).
- Clinical Frailty Scale (CFS) < 4 (25%).
- Serum albumin ≥ 2.5 g/dL (15%).
- BMI 16–40 kg/m² (10%).
- Age ≤ 70 years (10%).
- Cardiopulmonary clearance (10%), based on echocardiography and pulmonary function tests.
- Psychosocial suitability (10%), assessed using a structured social worker checklist.

Candidates achieving a composite score ≥ 75% were considered eligible. This design allowed high-MELD frail patients to be excluded, while lower-MELD robust patients could still qualify based on preserved reserve and favorable extrahepatic parameters.

### Frailty assessment

Frailty was assessed using the CFS, a validated 9-point scale (1 = “very fit,” 9 = “terminally ill”), with frailty defined as CFS ≥ 4 [9].

- Assessment team: three hepatologists, one physiotherapist, and one transplant nurse.
- Procedure: Independent evaluation of clinical records, physiotherapy assessments, and nursing documentation.
- Blinding: All raters were blinded to transplant eligibility decisions.
- Adjudication: Disagreements were resolved by consensus, with a senior hepatologist serving as adjudicator to ensure consistency, all evaluators underwent standardized training using published CFS anchors prior to assessments. In cases of discordant scoring, ratings were reviewed and adjudicated by a senior hepatologist until consensus was achieved. Due to logistical constraints, direct performance-based measures (e.g., grip strength, six-minute walk test) were not applied. Nevertheless, systematic documentation allowed consistent scoring across raters.

### Variables collected

- Demographics: age, sex, BMI.
- Hepatic severity: MELD, MELD-Na, Child-Pugh class.
- Complications of portal hypertension: ascites, hepatic encephalopathy, variceal bleeding.
- Laboratory parameters: albumin, bilirubin, INR, creatinine, sodium.
- Comorbidities: diabetes, hypertension, cardiovascular disease.

### Study size

- All eligible patients meeting inclusion criteria during the specified 9-month period were included. As this was an exploratory study, no formal sample size calculation was performed, and the analyses were intended to be hypothesis-generating rather than powered for definitive subgroup comparisons. Though the total cohort (*n* = 672) was sufficient to detect statistically significant differences in eligibility outcomes.

### Statistical analysis

All analyses were conducted using SPSS version 26.0 (IBM Corp., Armonk, NY). Descriptive statistics: Mean ± SD for normally distributed variables; median (IQR) for skewed distributions; frequencies (%) for categorical variables. Group comparisons: Student’s *t*-test or Mann–Whitney U for continuous variables; Chi-square or Fisher’s exact test for categorical variables. Correlations: Spearman’s rank correlation coefficients used to evaluate associations between CFS, MELD, albumin, and bilirubin. Multivariable logistic regression: Identified predictors of transplant eligibility, with odds ratios (OR) and 95% confidence intervals (CI). Interaction terms (MELD × CFS) were included to examine joint effects. Discriminative ability: Receiver Operating Characteristic (ROC) curves compared MELD vs. CFS. AUCs, sensitivity, specificity, PPV, NPV, and Youden’s Index were reported. Significance threshold: Two-tailed *p* < 0.05.

## Results

### Baseline characteristics and MELD stratification

A total of 672 patients with liver cirrhosis were evaluated for transplant eligibility during the study period. Among them, 36 patients (5%) had MELD scores ≥ 15, while the remaining 95% had MELD scores < 15. Patients in the higher MELD group exhibited significantly more advanced disease characteristics, including greater prevalence of diabetes (62% vs. 38%, *p* = 0.03), ascites (81% vs. 29%, *p* < 0.001), and higher bilirubin levels (2.8 ± 1.4 vs. 1.1 ± 0.7 mg/dL, *p* < 0.001). Notably, frailty burden was markedly elevated in this subgroup, with mean CFS scores of 6.9 ± 1.2 compared to 4.1 ± 1.8 in those with MELD < 15 (*p* < 0.001), despite comparable age and sex distribution (Table 1).

**Table 1:** Comparison between cases with MELD < 15 vs. ≥ 15

Characteristic	MELD < 15 (*n* ≈ 636)	MELD ≥ 15 (*n* = 36)	*p*-value
Age (mean ± SD)	60.5 ± 8.5	61.0 ± 9.0	0.75
Male (%)	80	78.6	0.85
Etiology (%)			0.2
- HCV	80	70	
- NBNC	15	25	
- HBV	5	5	
Diabetes (%)	38	62	0.03
Hypertension (%)	20	40	0.04
BMI (%)			0.1
- Normal	50	40	
- Overweight	30	20	
- Underweight	10	30	
- Obese	10	10	
Ascites (%)	29	81	< 0.001
Bilirubin (mean ± SD)	1.1 ± 0.7	2.8 ± 1.4	< 0.001
Albumin (mean ± SD)	3.7 ± 0.6	2.8 ± 0.5	< 0.001
CFS (mean ± SD)	4.1 ± 1.8	6.9 ± 1.2	< 0.001

*BMI* body mass index, *CFS* Clinical Frailty Scale, *MELD* Model for End-Stage Liver Disease, *SD* Standard deviation

### Correlations between frailty and liver disease severity

**Fig. 1:**
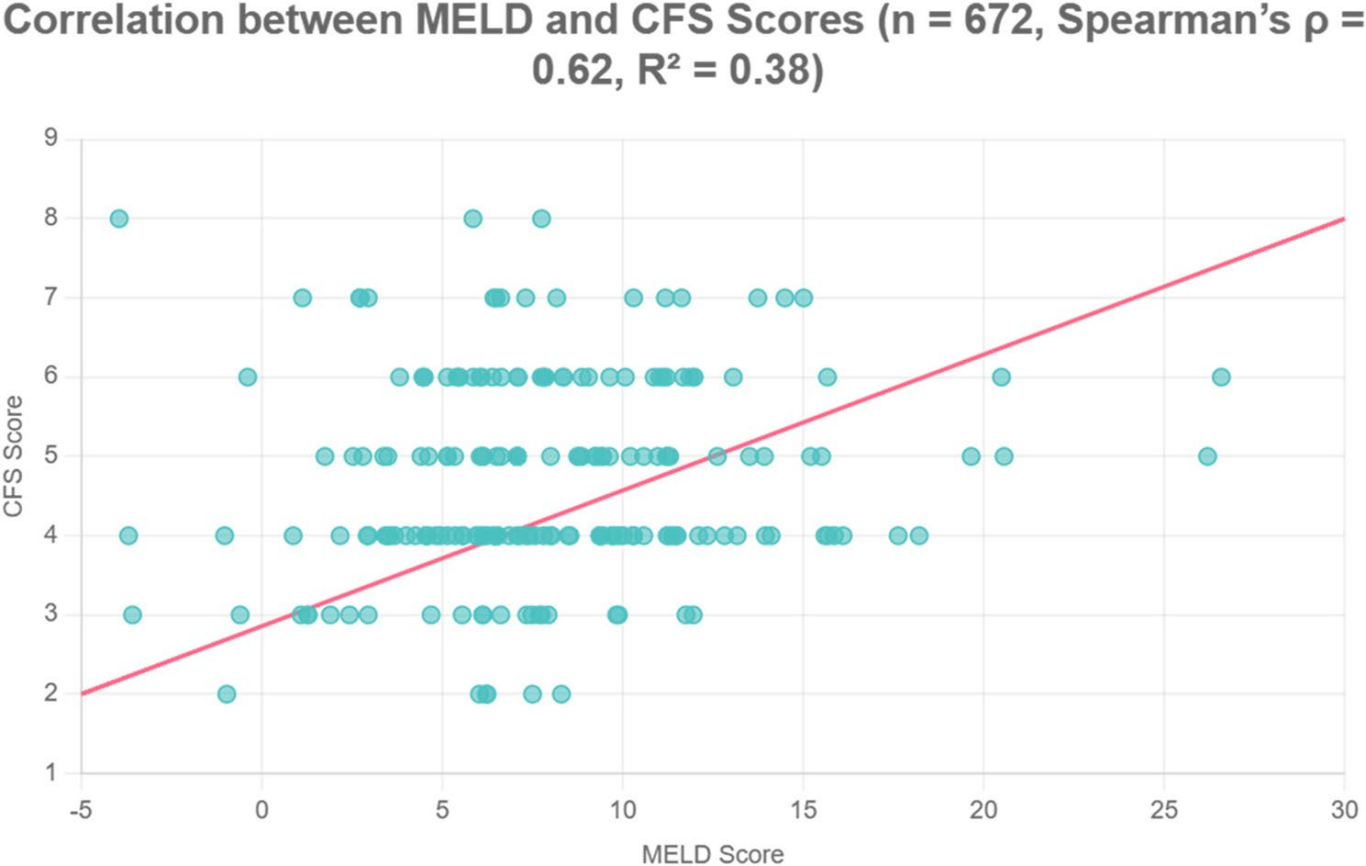
Scatter plot shows the correlation between MELD and CFS

Spearman’s correlation analysis revealed a moderate but significant association between CFS and MELD scores (*r* = 0.62, *p* < 0.001). Additional inverse correlations were observed between CFS and albumin (*r* = −0.57, *p* < 0.001), while bilirubin demonstrated a positive correlation with frailty scores (*r* = 0.49, *p* < 0.001) (Table 2). A moderate positive correlation is observed (*r* = 0.62, R² = 0.38). The regression line is displayed with 95% confidence intervals, demonstrating consistent association across the observed range of values (Fig. 1).

**Table 2:** Comparison between eligible and ineligible patients for transplantation

Parameter	Eligible (*n* = 76)	Ineligible (*n* = 596)	*p*-value
MELD score (mean ± SD)	8.20 ± 3.10	14.70 ± 4.50	< 0.001*
CFS ≥ 5 (%)	22.00	85.00	< 0.001*
Albumin < 3.5 g/dL (%)	29.00	77.00	< 0.001*
Ascites (%)	18.00	69.00	< 0.001*
Hypertension (%)	31.00	62.00	< 0.001*

*CFS* Clinical Frailty Scale, *SD* Standard deviation

### Transplant eligibility and frailty prevalence

**Fig. 2:**
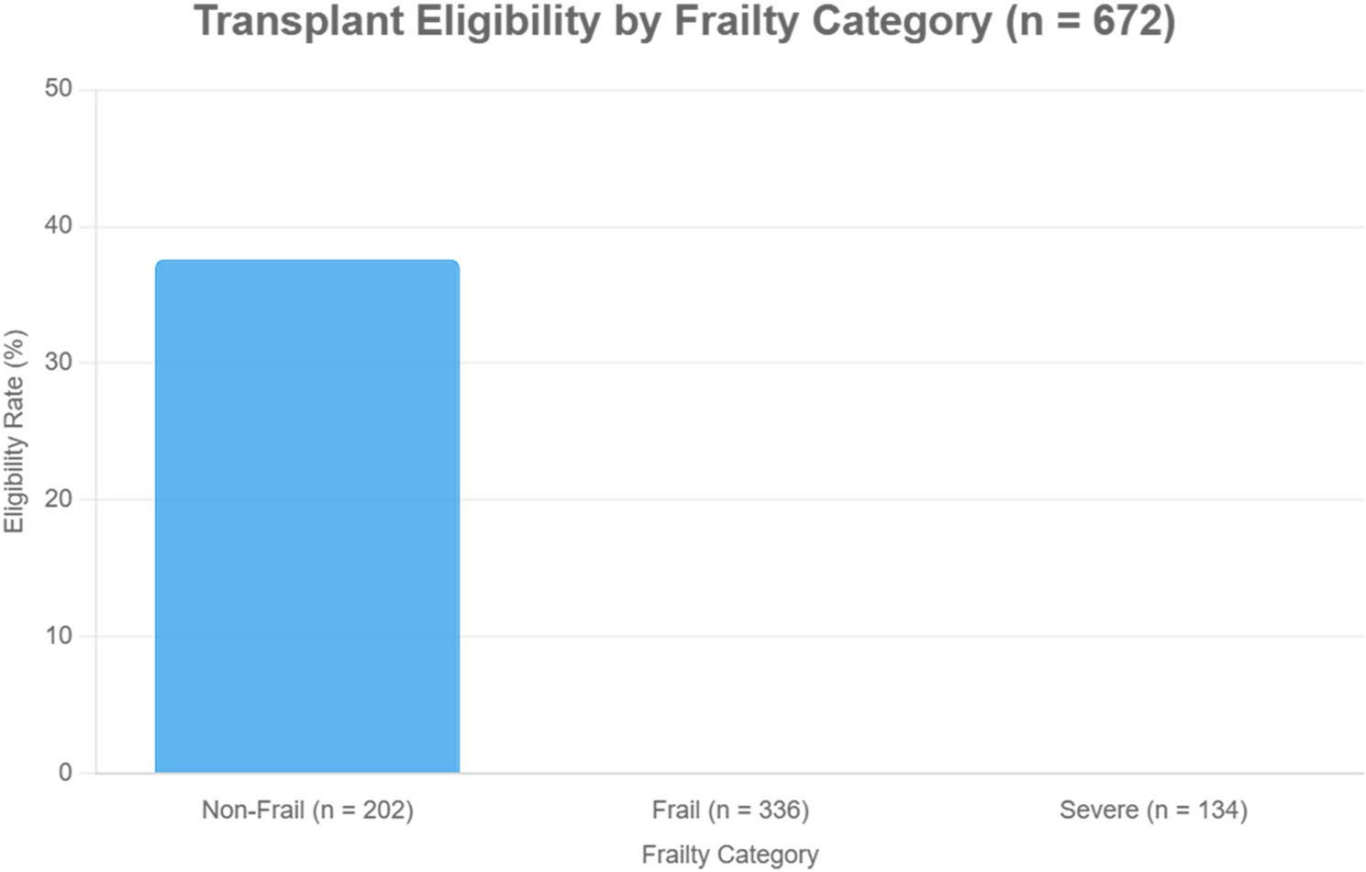
Distribution of frailty status among transplant-eligible and ineligible patients

Out of the full cohort, only 76 patients (11.3%) were deemed eligible for transplantation. Eligible individuals had significantly lower MELD scores (8.2 ± 3.1 vs. 14.7 ± 4.5, *p* < 0.001), less frequent ascites (18% vs. 69%, *p* < 0.001), and lower prevalence of hypertension (31% vs. 62%, *p* < 0.001). Strikingly, the presence of frailty (CFS ≥ 5) was observed in only 22% of eligible patients, compared to 85% among those deemed ineligible (*p* < 0.001) (Table 3) (Fig. 2).

**Table 3:** Correlations between frailty (CFS) and disease severity measures

Variable	Spearman’s *r*	*p*-value
MELD score	0.62	< 0.001*
Albumin	–0.57	< 0.001*
Bilirubin	0.49	< 0.001*

*CFS* Clinical Frailty Scale, *MELD* Model for End-Stage Liver Disease

### Comparative predictive accuracy of frailty vs. MELD

**Fig. 3:**
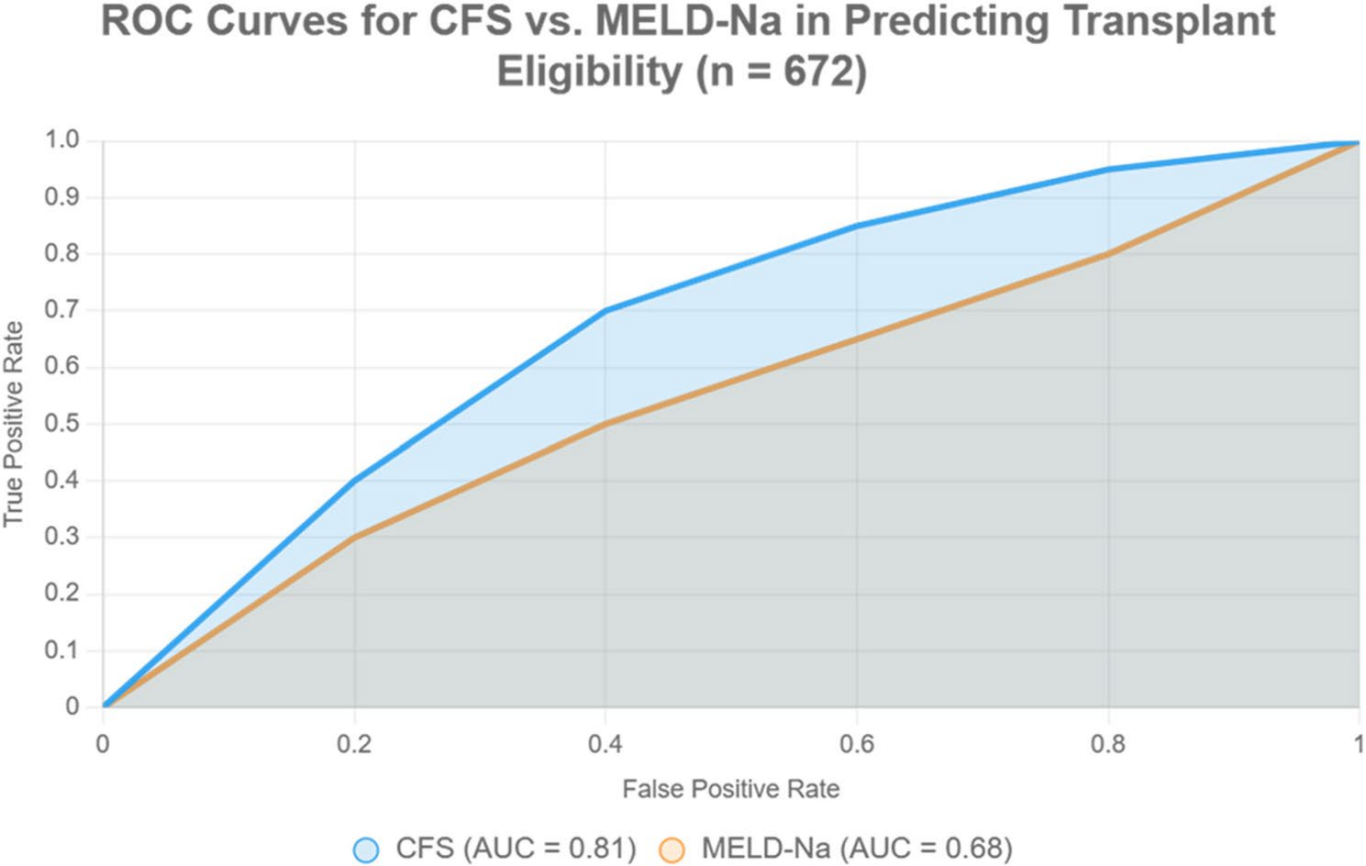
Predictive performance of clinical frailty scale (CFS) vs. MELD-Na for liver transplant eligibility

The ROC curve highlights CFS’s superior predictive performance over MELD-Na (AUC: 0.81 vs. 0.68, sensitivity: 92% vs. 76%, specificity: 88% vs. 64%), supporting the manuscript’s recommendation to use CFS in transplant eligibility assessments (Table 4- Fig. 3).

**Table 4:** Predictive performance of frailty (CFS) vs. MELD for transplant eligibility

Metric	CFS Score	MELD Score	*p*-value (difference)
AUC (95% CI)	0.81 (0.76–0.86)	0.68 (0.62–0.74)	0.003*
Optimal cutoff	≥ 4	≥ 15	–
Sensitivity (%)	92.00	76.00	< 0.001*
Specificity (%)	88.00	64.00	< 0.001*
PPV (%)	83.00	58.00	< 0.001*
NPV (%)	94.00	80.00	0.002*
Youden’s Index (J)	0.80	0.40	< 0.001*

*AUC* Area under ROC curve, *CI* Confidence interval, *CFS* Clinical Frailty Scale, *MELD* Model for End-Stage Liver Disease, *NPV* Negative predictive value, *PPV* Positive predictive value

### Predictors of transplant eligibility

In multivariate logistic regression analysis, the CFS emerged as the strongest independent predictor of transplant ineligibility (OR = 0.38, 95% CI: 0.29–0.51, *p* < 0.001), even after adjusting for MELD score, age, ascites, and comorbidities. Higher MELD score was also significantly \associated with reduced eligibility (OR = 0.88, 95% CI: 0.82–0.95, *p* = 0.001). Ascites independently predicted ineligibility (OR = 2.15, 95% CI: 1.21–3.83, *p* = 0.009), whereas age, diabetes, and hypertension were not statistically significant predictors in the final model (Table 5).

**Table 5:** Logistic regression predictors of transplant eligibility

Predictor	Odds Ratio	95% CI	*p*-value
CFS score	0.38	0.29–0.51	< 0.001*
Age (per year)	0.97	0.93–1.01	0.12
MELD score	0.88	0.82–0.95	0.001*
Diabetes	1.42	0.79–2.55	0.24
Hypertension	1.18	0.67–2.08	0.57
Ascites	2.15	1.21–3.83	0.009*

*CI* Confidence interval, *CFS* Clinical Frailty Scale, *MELD* Model for End-Stage Liver Disease, *OR* Odds ratio

## Discussion

This prospective cohort study demonstrates that frailty, as measured by CFS, is a decisive determinant of liver transplant eligibility, surpassing MELD score in predictive performance. Each 1-point increase in CFS was associated with a 62% reduction in the odds of eligibility, and ROC analyses confirmed its superior discriminative accuracy (AUC 0.81 vs. 0.68).

Our study utilized an exploratory eligibility framework in which frailty (CFS) was assigned a 25% weighting and MELD score a 30% weighting. We acknowledge that this artificial construct does not reflect current organ allocation principles, where MELD-based urgency remains the dominant determinant of access to transplantation. The rationale for this design was methodological: to create a standardized model that would allow us to quantify the relative contribution of frailty alongside disease severity in shaping transplant eligibility.

This approach produced paradoxical patterns, with some patients with higher MELD scores deemed ineligible due to severe frailty, while others with lower MELD but preserved physiological reserve remained eligible. The observed moderate correlation between MELD and CFS (*r* = 0.62) highlights the partly overlapping yet distinct constructs captured by these measures. Discordant cases provide particularly valuable insights: patients with high MELD but low frailty scores often represent individuals with acute or rapidly progressive hepatic decompensation but preserved functional reserve, whereas those with low MELD but high frailty typically reflect patients with compensated cirrhosis complicated by sarcopenia, malnutrition, or comorbid conditions. These phenotypes illustrate how MELD alone may underestimate risk in frail but biochemically stable patients, while CFS may provide little additional value in acutely decompensated patients with already high MELD scores. Rather than contradicting established transplant principles, this finding underscores an important tension between medical urgency (reflected by MELD) and physiological suitability (captured by frailty status). These paradoxes align with clinical reality, where transplant teams frequently exclude patients with advanced frailty despite high MELD scores, due to concerns about perioperative risk, poor recovery, and diminished post-transplant survival.

Our framework should therefore be interpreted as exploratory and hypothesis-generating, not as a proposal to replace MELD-based allocation. It highlights that frailty represents an independent determinant of transplant candidacy, consistent with prior evidence showing frailty predicts waitlist mortality, post-transplant complications, and overall survival ( [4]– [5]). Kremer et al. subsequently validated CFS as a predictor of mortality in cirrhotic patients [6], while Lai et al. (2014) established the Fried Frailty Index (LFI) as an independent predictor of waitlist death [7]. The American Society of Transplantation (AST) and other expert consensus groups have already advocated for the incorporation of frailty into pretransplant evaluation, not as a substitute but as a complementary dimension of risk stratification [8]. Although that panel favored the LFI, our reliance on the CFS reflects its practicality and feasibility in resource-constrained environments, consistent with recent pragmatic validations.

Importantly, the predominance of low-MELD patients in our cohort highlights a subgroup in which MELD alone inadequately reflects clinical vulnerability. Prior evidence demonstrated that patients with MELD < 15 may still experience substantial morbidity and waitlist mortality, with frailty serving as an independent predictor of poor outcomes in this population [7]. Our findings therefore underscore frailty’s complementary value precisely where biochemical criteria are least informative and support the need for multicenter validation across the full MELD spectrum.

More recent work underscores the relevance of our findings. Tang et al. (2025) synthesized global evidence confirming frailty as a robust predictor of adverse transplant outcomes [10], while Pahari et al. (2025) emphasized the imperative of embedding frailty into integrative candidate assessment strategies [11]. By prospectively applying frailty to eligibility determination in a predominantly low-MELD cohort, our study not only corroborates these trajectories but also advances them—providing one of the first prospective demonstrations of frailty’s utility as a determinant of candidacy, rather than solely as a prognostic adjunct.

The clinical significance of these results is multifaceted. First, reliance on MELD alone risks overestimating candidacy in frail patients with advanced disease and underestimating it in robust patients with low MELD. Incorporating frailty corrects this imbalance, ensuring candidates with meaningful physiologic reserve are not overlooked. Second, unlike MELD, frailty is potentially modifiable. Interventions such as structured prehabilitation—including nutrition, resistance training, and physical activity—have been shown to reverse frailty in cirrhosis and improve transplant readiness ( [12]– [13]). Third, frailty-based assessment aligns allocation with both ethical imperatives and resource efficiency by prioritizing patients who are most likely to achieve survival benefit and quality-of-life restoration after transplantation.

The significant correlations between frailty and biochemical markers such as albumin and bilirubin likely reflect convergent pathophysiology rather than independent associations. Hypoalbuminemia denotes impaired hepatic synthesis and malnutrition, while hyperbilirubinemia reflects cholestasis and hepatocellular dysfunction—processes intrinsically linked to sarcopenia, systemic inflammation, and protein–energy wasting, all central to frailty in cirrhosis. Thus, these correlations should be interpreted as overlapping biological pathways, underscoring the need for advanced modeling to delineate independent prognostic contributions [14].

This study contributes several novel dimensions. It is the first prospective investigation to directly compare CFS against MELD for transplant eligibility decisions, moving beyond prognostic prediction to candidate selection itself. By focusing on a predominantly low-MELD cohort (95% <15), it offers rare insight into early-stage cirrhosis populations often underrepresented in the literature. The use of a structured, weighted eligibility framework marks an innovative attempt to standardize decision-making beyond subjective committee consensus. Finally, this study provides unique regional data from a Middle Eastern/North African cohort, enriching the global evidence base that has thus far been dominated by Western populations.

However, this study has several limitations that merit acknowledgment. The weighted eligibility framework—assigning fixed contributions to frailty and MELD—was intentionally exploratory, designed to illuminate their relative influence, and should be regarded as hypothesis-generating rather than prescriptive. The deliberate exclusion of extra-MELD indications reduced heterogeneity and enabled focused assessment of MELD-driven candidacy, but necessarily limited external validity, as such indications comprise a substantial proportion of real-world transplant listings. Furthermore, the 9-month observation period may not fully account for potential seasonal variability in patient referral patterns or disease severity, although institutional audit data suggest relatively stable referral volumes across the year. Larger, multicenter studies with extended follow-up will be required to confirm these observations. Our analysis was restricted to pretransplant eligibility determinations, without linkage to post-transplant survival or graft outcomes, preventing evaluation of long-term clinical benefit. The characteristics of the cohort—predominantly low MELD (< 15), a modest eligibility rate (11.3%), and derivation from a single Egyptian center with high HCV prevalence—further constrain generalizability to advanced disease and to regions where NAFLD or alcohol-related liver disease predominate. Frailty assessment relied on the Clinical Frailty Scale rather than the AST-endorsed LFI, and inter-rater reliability was not formally measured, raising concerns about reproducibility. Additionally, MELD was analyzed dichotomously (< 15 vs. ≥ 15), an approach that may obscure subtler associations across the disease continuum, while the limited sample size and short follow-up reduced power for subgroup and temporal analyses.

Future research should validate these findings in larger, multicenter cohorts spanning the full spectrum of MELD and extra-MELD indications. Standardization of frailty assessment, ideally combining pragmatic tools such as CFS with objective physical measures, will be essential for reproducibility. Interventional studies targeting frailty reversal through prehabilitation could establish whether eligibility and outcomes can be systematically improved. Ultimately, incorporation of frailty into intention-to-treat allocation models, balancing waitlist and post-transplant survival, will clarify its role in optimizing both fairness and efficiency in organ allocation. Moving forward, prospective multicenter studies are required to validate weighting systems that integrate both MELD and frailty, ideally linked to post-transplant outcomes. Until then, our findings should be viewed as supportive evidence for incorporating frailty into routine assessments of transplant candidacy, rather than as a direct model for allocation policy.

## Conclusion

This study provides the first prospective evidence that frailty can directly inform transplant eligibility, outperforming MELD-Na and exposing hidden vulnerability in low-MELD patients. By moving frailty from a prognostic marker to a determinant of candidacy within a structured framework, our findings represent a novel step toward integrating functional status into equitable, patient-centered transplant decision-making.

## Data Availability

“Data is available upon request from the corresponding author.”

## Abbreviations

CFS: Clinical Frailty Scale
MELD: Model for End-Stage Liver Disease
ALBI: Albumin-Bilirubin score
CPU: Child-Pugh score
LFI: Liver frailty index
BMI: Body Mass Index
AUC: area under ROC curve
CI: confidence interval
NAFLD: Non-alcoholic fatty liver disease
NPV: negative predictive value
PPV: positive predictive value
